# DNA‐PKcs/AKT1 inhibits epithelial–mesenchymal transition during radiation‐induced pulmonary fibrosis by inducing ubiquitination and degradation of Twist1

**DOI:** 10.1002/ctm2.1690

**Published:** 2024-05-17

**Authors:** Ziyan Yan, Jiaojiao Zhu, Yuhao Liu, Zhongqiu Li, Xinxin Liang, Shenghui Zhou, Yifan Hou, Huixi Chen, Lin Zhou, Ping Wang, Xingkun Ao, Shanshan Gao, Xin Huang, Ping‐Kun Zhou, Yongqing Gu

**Affiliations:** ^1^ Beijing Key Laboratory for Radiobiology Beijing Institute of Radiation Medicine Beijing China; ^2^ State Key Laboratory of Stem Cell and Reproductive Biology Institute of Zoology Chinese Academy of Sciences Beijing China; ^3^ Hengyang Medical College University of South China Hengyang China; ^4^ College of Life Sciences Hebei University Baoding China

**Keywords:** DNA‐PKcs, epithelial–mesenchymal transition, radiation‐induced pulmonary fibrosis, Twist1, VND3207

## Abstract

**Introduction:**

Radiation‐induced pulmonary fibrosis (RIPF) is a chronic, progressive, irreversible lung interstitial disease that develops after radiotherapy. Although several previous studies have focused on the mechanism of epithelial–mesenchymal transition (EMT) in lung epithelial cells, the essential factors involved in this process remain poorly understood. The DNA‐dependent protein kinase catalytic subunit (DNA‐PKcs) exhibits strong repair capacity when cells undergo radiation‐induced damage; whether DNA‐PKcs regulates EMT during RIPF remains unclear.

**Objectives:**

To investigate the role and molecular mechanism of DNA‐PKcs in RIPF and provide an important theoretical basis for utilising DNA‐PKcs‐targeted drugs for preventing RIPF.

**Methods:**

DNA‐PKcs knockout (DPK^−/−^) mice were generated via the Cas9/sgRNA technique and subjected to whole chest ionizing radiation (IR) at a 20 Gy dose. Before whole chest IR, the mice were intragastrically administered the DNA‐PKcs‐targeted drug VND3207. Lung tissues were collected at 1 and 5 months after IR.

**Results:**

The expression of DNA‐PKcs is low in pulmonary fibrosis (PF) patients. DNA‐PKcs deficiency significantly exacerbated RIPF by promoting EMT in lung epithelial cells. Mechanistically, DNA‐PKcs deletion by shRNA or inhibitor NU7441 maintained the protein stability of Twist1. Furthermore, AKT1 mediated the interaction between DNA‐PKcs and Twist1. High Twist1 expression and EMT‐associated changes caused by DNA‐PKcs deletion were blocked by insulin‐like growth factor‐1 (IGF‐1), an AKT1 agonist. The radioprotective drug VND3207 prevented IR‐induced EMT and alleviated RIPF in mice by stimulating the kinase activity of DNA‐PKcs.

**Conclusion:**

Our study clarified the critical role and mechanism of DNA‐PKcs in RIPF and showed that it could be a potential target for preventing RIPF.

## Graphical abstract

1. DNA‐PKcs deficiency accelerates the process of EMT by inhibiting AKT1 phosphorylation, thus stabilising Twist1, which ultimately contributes to RIPF.
2. The radioprotective drug VND3207 effectively alleviates RIPF by targeting DNA‐PKcs to prevent radiation‐induced EMT.
3. This study revealed a novel pathway, DNA‐PKcs/AKT1/Twist1 as a crucial signalling pathway for RIPF.

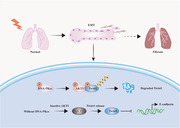

## 1 INTRODUCTION

Radiation‐induced lung injury (RILI) includes early radiation‐induced pneumonia (RP) and late radiation‐induced pulmonary fibrosis (RIPF). RIPF is a progressive, irreversible chronic lung disease characterised by damage to alveolar epithelial cells, excessive proliferation and activation of fibroblasts, and massive deposition of extracellular matrix (ECM), which leads to severe physiological abnormalities and chronic respiratory failure.^1^ The abnormal proliferation and activation of fibroblasts are key factors that greatly favour pulmonary fibrosis (PF). Alveolar epithelial cells are target cells that directly respond to ionising radiation (IR).^2^, ^3^, ^4^ Type II alveolar epithelial cells (AECII) are stem cells that can proliferate and differentiate into type I alveolar epithelial cells (AECI) to preserve the alveolar structure. Furthermore, epithelial‒mesenchymal transition (EMT) occurs in irradiated AECII, resulting in the formation of fibroblasts or cells with morphology similar to that of fibroblasts. Previous study has shown that approximately 30%−50% of mouse lung fibroblasts come from EMT of local epithelium.^5^ Therefore, the irradiated alveolar epithelial cells undergoing EMT serve as the main sources of myofibroblasts in the RIPF^5^, ^6^, ^7^, ^8^, ^9^, ^10^, ^11^; however, the mechanism of IR‐induced alveolar epithelial EMT in RIPF remains unclear.

DNA‐dependent protein kinase catalytic subunit (DNA‐PKcs) belongs to the PI3K‐related enzyme family and is a key enzyme involved in DNA damage repair.^12^ DNA‐PKcs is a DNA double‐strand break (DSB) sensor. DNA‐PKcs is a catalytic subunit that functions as a component of the non‐homologous end joining repair pathway. It forms a DNA‐dependent protein kinase (DNA‐PK) complex with the regulatory subunit Ku70/80.^13^, ^14^, ^15^ DNA‐PKcs plays a dominant role in the regulation of DNA damage in response to IR. There are many outcomes following DNA damage induced by IR.^16^ In certain instances, DNA damage can lead to the induction of EMT.^17^, ^18^ Although DNA‐PKcs can participate in alveolar epithelial EMT remains unclear. Study has documented that DNA‐PKcs could drive the progression of fibrosis in chronic kidney disease (CKD) by regulating the TAF7/mTORC1 signalling pathway.^19^ However, idiopathic pulmonary fibrosis (IPF) lung tissues showed a decrease in DNA‐PKcs expression.^20^ DNA‐PKcs had functional differences in physiological and pathological environments and in different tissues and cells, and its role in fibrosis is controversial.

In the present study, we investigated whether DNA‐PKcs deficiency facilitates RIPF by regulating EMT in AECIIs. We found that the deletion of DNA‐PKcs promoted Twist1 expression in vitro and in vivo. Furthermore, DNA‐PKcs regulates the ubiquitination and degradation of Twist1 by inhibiting AKT1 phosphorylation. Additionally, the radioprotective agent VND3207 can target DNA‐PKcs and exert a protective effect on RIPF. This study provides compelling evidence revealing the role of the DNA‐PKcs/AKT1/Twist1 axis in the regulation of IR‐induced EMT and provides a molecular and theoretical basis for treating RIPF.

## 2 MATERIALS AND METHODS

### 2.1 Generation and validation of DNA‐PKcs knockout mice

DNA‐PKcs knockout (DPK^−/−^) mice were purchased from Bestcell Model Bio‐Tech Co., Ltd. Wild‐type (WT) mice were provided by SPF Biotechnology Co., Ltd. All the mice with a C57BL/6J background. The knockout efficiency of DNA‐PKcs was verified by genotyping the two DNA sequencing and Western blotting, and the required data were provided by Tsingke Biotechnology Co., Ltd. (Figure S1). Following primers were used: primer 1—forward primer, 5ʹ‐CAT TTG CGT TGT CCC GAG TG‐3ʹ; reverse primer, 5ʹ‐GAC GTT GAC TAC ACG CAC CA‐3ʹ; primer 2—forward primer, 5ʹ‐GCC AGC GCA TAG TGA GAA CT‐3ʹ; and reverse primer, 5ʹ‐TCT GCA AGC AAG TTT CTG GGA‐3ʹ. Mice were reared in the Animal Center of the Academy of Military Medical Sciences under the following conditions: standard diet; temperature, 22 ± 2°C; humidity, 60%; light‒dark (12 h:12 h) cycle.

### 2.2 Mouse radiation and VND3207 treatment

WT and DPK^−/−^ mice (8−10 weeks old, 50% male mice and 50% female mice) were used to establish the RIPF model by whole chest IR with ^60^Co γ‐rays at a 20 Gy dose, and the mice without IR were taken as negative control (NC). The mice were assigned to four groups using their weight as a guide, namely, the WT + NC, WT + IR, DPK^−/−^ + NC, and DPK^−/−^ + IR groups, and their lung tissues were collected at 1 and 5 months after IR. To investigate the effect of the vanillin derivative VND3207 on RIPF, the mice were administered 100 mg/kg VND3207 (dissolved in 5% CMC‐Na) by gavage 30 min before IR, and the same volume of 5% CMC‐Na was administered as a control. CMC‐Na and VND3207 were obtained from our laboratory.

### 2.3 Pathological staining and histological analysis

Haematoxylin‒eosin (H&E) staining was used to examine the histopathology of mouse lung tissues (Cat# ZLI‐9610, ZSGB‐BIO), and the results were quantified by a semiquantitative scoring system reported by Szapiel. Masson's trichrome staining (Cat# G1340, Solarbio Life Science) was used to detect collagen content in lung tissues, and collagen quantification was performed using ImageJ software.

### 2.4 Real‐time qPCR

Total RNA was isolated from mouse lung tissues using TRIzol reagent (Invitrogen). Using NanoDrop 2000 spectrophotometer (Thermo Scientific) was used to estimate RNA quantity and quality. cDNA synthesis was performed using RT SuperMix for qPCR (Cat# R323, Vazyme). Real‐time quantitative PCR (RT‐qPCR) analysis was conducted in triplicate using SYBR Mix (Cat# Q712, Vazyme). The primer sequences are listed in Table S1.

### 2.5 Cell culture and treatment

A549 cells and MLE‐12 cells were preserved in our laboratory (National Collection of Authenticated Cell Cultures). A549‐shDNA‐PKcs and A549‐shNC cells were generated from A549 cells by transfecting the cells with lentiviruses carrying shRNA‐DPKcs‐U6/puromycin and shRNA‐NC vectors. The lentiviruses synthesised from GenePharma, and sequences shown as follow: shRNA‐DNA‐PKcs: 5ʹ‐GGG CGC TAA TCG TAC TGA A‐3ʹ; shRNA‐NC: 5ʹ‐TTC TCC GAA CGT GTC ACG T‐3ʹ. Cells were cultured at 37°C and 5% CO_2_, supplemented with high‐glucose Dulbecco's Modified Eagle's Mudium (DMEM) containing 10% foetal bovine serum (Cat# FSP500, ExCell Bio) for nutrient, and 1% penicillin‒streptomycin solution (Cat# 2106030 M, Rongxia) was added to prevent cell contamination. Cells were irradiated with ^60^Co γ‐rays at a dose of 6 Gy. For the VND3207 treatment group, the cells were first treated with 40 μM VND3207 (dissolved in dimethyl sulfoxide (DMSO)) for 2 h before IR.

### 2.6 Cell transfection

Plasmid DNA and siRNA (GenePharma) were transfected into cells using Lipofectamine 2000 (Invitrogen). The HA‐Ub plasmid, which increases the ubiquitination level of intracellular proteins, was constructed in our laboratory and used to detect Twist1 ubiquitination. Twist1‐specific siRNA targeted the following sequence in Twist1 mRNA: 5ʹ‐GCU GAG CAA GAU UCA GAC CTT‐3ʹ. The following sequence of human DNA‐PKcs‐specific siRNA was used to target DNA‐PKcs mRNA: 5ʹ‐GGG CGC TAA TCG TAC TGA A‐3ʹ. A mouse DNA‐PKcs‐specific siRNA targeting the following sequence of DNA‐PKcs mRNA was used: 5ʹ‐GCUAGCGACCGACAAACUATT‐3ʹ.

### 2.7 Co‐immunoprecipitation

The cells were lysed on ice using lysis buffer (50 mM Tris‒HCl [pH 8.0], 300 mM NaCl, .5% [v/v] NP‐40, 1 mM EDTA) supplemented with protease inhibitor cocktail and phosphokinase inhibitor (Roche). Following with the supernatants incubated with 1 μg of primary antibodies for 2 h, protein A/G agarose was added overnight incubation at 4°C. After washing the agarose beads four times with lysis buffer, immunoprecipitation complexes were collected and analysed by Western blotting.

### 2.8 Protein stability assay

The protein stability was detected by cycloheximide (CHX) chase assay and in vivo ubiquitination assay. The shNC cells and shDPK cells were treated with 100 μg/mL CHX (Sigma‒Aldrich) for the indicated hours. Meanwhile, A549 cells were treated with CHX prior to subsequent treatment with 10 μM NU7441 (Selleck) or DMSO for 6 h. The cells were transfected with HA‐Ub plasmid and incubated for 24 h before exposure to 10 μM MG132 (Sigma‒Aldrich) for 6 h to inhibit proteasome activity. Protein expression levels were measured by Western blotting assay.

### 2.9 Western blotting assay

Total proteins were extracted with commercial protein extraction reagents (Cat# 78501 and Cat# 78510, Thermo). The target proteins expression was determined by the routine Western blotting analysis, and the primary antibodies used in the experiment are shown in Table S2. Immunoreactive bands in three independent experiments were quantified by densitometry using ImageJ software.

### 2.10 Immunofluorescence staining

After deparaffinisation, hydration and washing, the paraffin sections were subjected to high‐pressure antigen retrieval using citrate buffer (pH 6.0). The Triton buffer (.25% Triton X‐100 in phosphate‐buffered saline) used to permeate sections. After blocking the sections with goat serum, the primary antibodies were incubated overnight at 4°C, and the secondary antibodies were incubated for 1 h at room temperature the following day. Sections were mounted with the medium containing 4',6‐diamidino‐2‐phenylindole (DAPI) for fluorescence (Cat# ZLI‐9557, ZSGB‐BIO). A same operation was performed on cells. Images were scanned with a confocal immunofluorescence microscope (Carl Zeiss). The primary antibodies used in the experiment are shown in Table S2.

### 2.11 Immunohistochemical staining

After deparaffinisation, hydration and washing, the paraffin sections were subjected to high‐pressure antigen retrieval using citrate buffer (pH 6.0). According to the instructions, endogenous peroxidase activity was neutralised with blocking agents after cooling to room temperature. After blocking the sections with goat serum, the primary antibodies were incubated overnight at 4°C, and the secondary antibodies were incubated for 1 h at room temperature the following day, followed by DAB colour development. Nuclei were stained with haematoxylin. Images were scanned with a pathological microscope (Nikon). Antibodies used are shown in Table S2.

### 2.12 Gene expression data from the public database

To analyse gene expression profiles in PF, independent gene expression microarray datasets related to PF, including those of patients with PF (GSE21369) and mice with RIPF (GSE41789), were retrieved and downloaded from the GEO database (https://www.ncbi.nlm.nih.gov/geo).

### 2.13 Statistical analysis

All experiments were performed with at least three replicates in each assay or with three independent experiments. SPSS 25.0 (IBM) or GraphPad Prism was used to the statistical analyses and mapping. All the data are shown as the mean ± standard error of the mean. Unpaired *t*‐test and analysis of variance were used for comparisons between two or more groups, respectively. *p *< .05 was considered statistically significant.

## 3 RESULTS

### 3.1 DNA‐PKcs deficiency aggravated RILI

**FIGURE 1:**
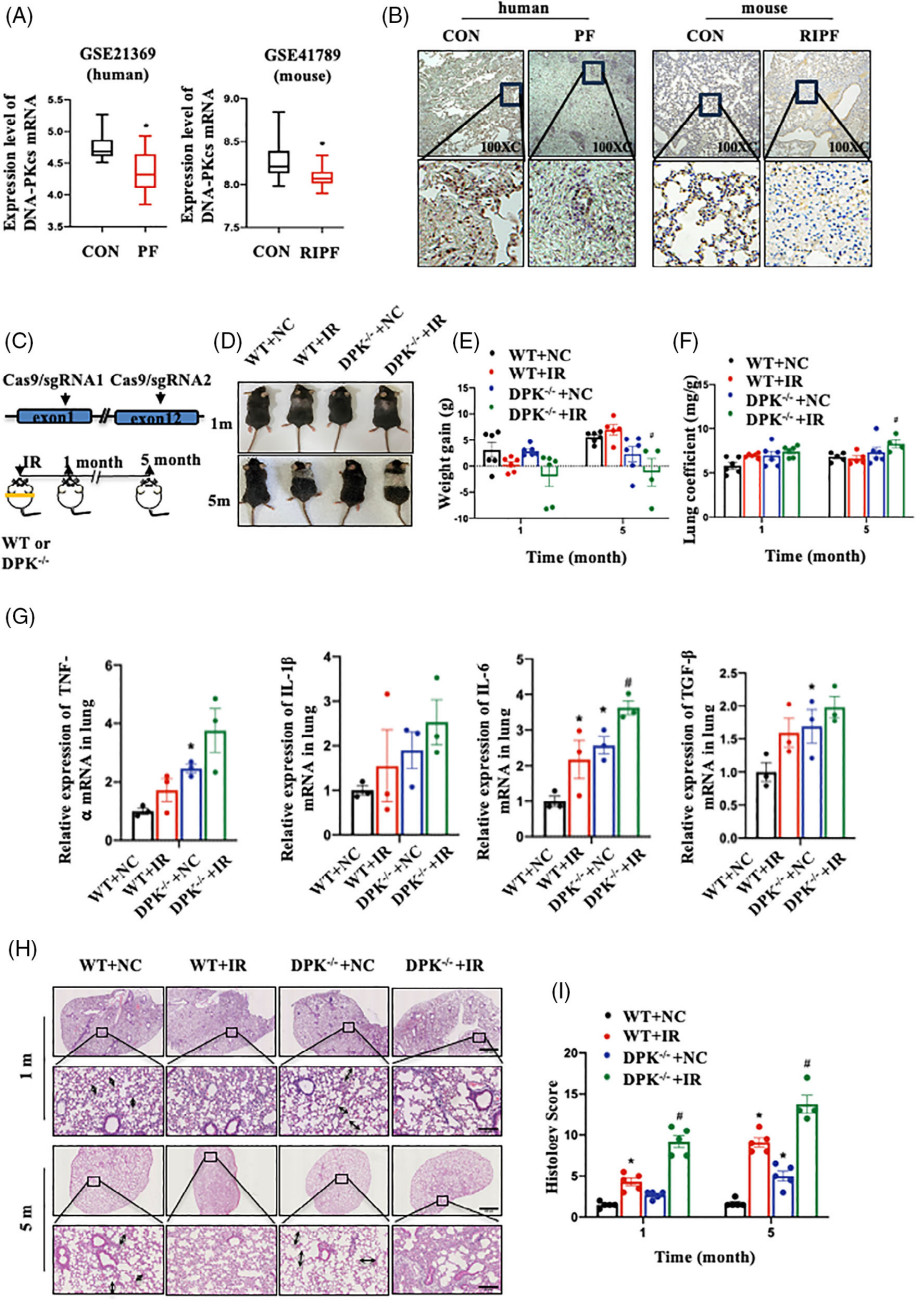
DNA‐dependent protein kinase catalytic subunit (DNA‐PKcs) deficiency aggravated radiation‐induced lung injury (RILI). (A) DNA‐PKcs expression in patients with pulmonary fibrosis (GSE21369) and mice with radiation‐induced pulmonary fibrosis (GSE41789). (B) DNA‐PKcs expression in patients with pulmonary fibrosis and mice with radiation‐induced pulmonary fibrosis were detected by immunohistochemical staining. (C) DNA‐PKcs knockout strategy and IR regimen in mice. (D) General condition of the mice at 1 and 5 months after whole chest IR at a 20 Gy dose. (E) Changes in the body weight of mice at 1 and 5 months after IR (*n* = 4−6 mice per group). (F) Lung coefficient at 1 and 5 months after IR (*n* = 4−6 mice per group). (G) Relative mRNA expression of TNF‐α, IL‐1β, IL‐6 and TGF‐β in lung tissues at 1 month after IR (*n* = 3 mice per group). (H) Haematoxylin‒eosin (H&E) staining of lung tissues at 1 and 5 months after IR. The length of the double arrow line segment indicates the size of the alveolar cavity. Scale bar: 300 or 80 μm. (I) Semi‐quantitative scoring results of lung pathology (*n* = 4 or 5 mice per group). Data are expressed as the means ± standard error of the mean (SEM); ^*^ *p* < .05, compared with the WT + NC group; ^#^ *p *< .05, compared with the WT + IR group. WT, wild type.

The RNA‐seq data of lung tissues from patients with PF and mice with RIPF (Figure 1A) showed decreased expression of DNA‐PKcs. We then confirmed the expression and activity of DNA‐PKcs in human PF and RIPF mouse tissue and found that its expression and activity (DNA‐PKcs ser2056) decreased (Figures 1B and S1A,B). Considering DNA‐PKcs' function in DSB damage repair, we further examined the co‐localisation of DNA‐PKcs ser2056 and γ‐H2AX in a RIPF mouse model. Compared to those in normal mice, the DNA‐PKcs ser 2056 decreased and γ‐H2AX increased the lung tissues of RIPF mice. Moreover, DNA‐PKcs ser2056 and γ‐H2AX co‐localising regions did not show an obvious increase in RIPF, which indicated an increase in the DNA damage in RIPF (Figure S1C,D). To assess how DNA‐PKcs affects RILI, DNA‐PKcs knockout mice were generated using the CRISPR‐Cas9 technique, and DNA sequencing and Western blotting were used to confirm successful knockout. Mice were exposed to whole chest IR at a dose of 20 Gy (Figures 1C and S2). At 1 and 5 months post‐IR, the mice were observed for symptoms of early RP and late RIPF, respectively. Compared to WT mice, DNA‐PKcs^−/−^ mice exhibited more significant hair loss and whitening in the irradiated area (Figure 1D) and greater weight loss (Figure 1E) after IR; these findings indicated that DNA‐PKcs deficiency induced severe radiation damage in these mice. Compared with that of WT mice, the lung coefficient (lung weight/body weight) of DNA‐PKcs^−/−^ mice was significantly greater after IR; this finding indicated that more severe pulmonary oedema was induced by IR after DNA‐PKcs deletion (Figure 1F). Furthermore, because of DNA‐PKcs deficiency, lung tissues had noticeably higher expression levels of proinflammatory cytokines such as tumour necrosis factor‐alpha (TNF‐α), interleukin‐1 beta (IL‐1β), interleukin‐6 (IL‐6) and transforming growth factor‐beta (TGF‐β) after IR (Figure 1G). Consistent with these findings, H&E staining revealed that the lung tissues of DNA‐PKcs^−/−^ mice were abnormally dilated, and more severe lung injury occurred after IR (Figure 1H,I); this condition was characterised by thickening and fragmentation of the lung septum, accompanied by extensive fibrin exudation, apparent inflammatory infiltration near the bronchi and congestion of small vessels. These observations show that DNA‐PKcs is critical for RILI.

### 3.2 DNA‐PKcs deficiency promoted IR‐induced collagen deposition in lung tissues

**FIGURE 2:**
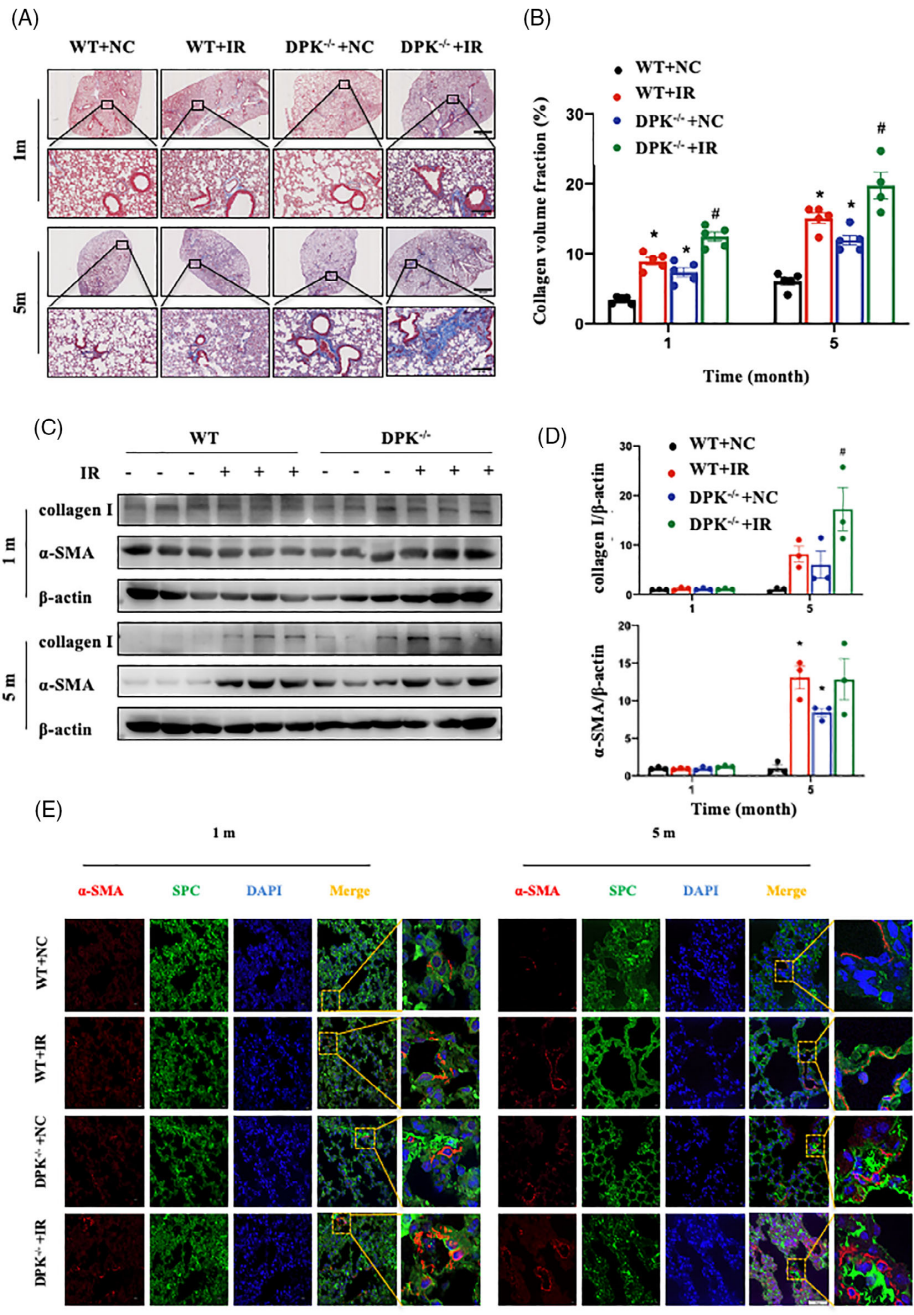
DNA‐dependent protein kinase catalytic subunit (DNA‐PKcs) deficiency promoted irradiation (IR)‐induced collagen deposition in lung tissues. (A) Masson's trichrome staining of lung tissues at 1 and 5 months after IR. Scale bar: 300 or 80 μm. (B) Collagen quantification results of lung tissues (*n* = 4 or 5 mice per group). Data are expressed as the means ± standard error of the mean (SEM); ^*^ *p* < .05, compared with the WT + NC group; ^#^ *p *< .05, compared with the WT + IR group. (C) Relative protein levels of collagen I and α‐SMA in lung tissues at 1 and 5 months after IR. Three mice per group were used for Western blotting analysis. (D) The expression of proteins in (C) was quantified. Data are expressed as the means ± SEM; ^*^ *p* < .05, compared with the WT + NC group; ^#^ *p *< .05, compared with the WT + IR group. (E) The localisation of α‐SMA (shown in red) and SPC (shown in green) in lung tissues at 1 and 5 months after IR was analysed by confocal microscopy. Cell nuclei were visualised by DAPI (shown in blue). Scale bar: 50 μm. WT, wild type.

To assess the role of DNA‐PKcs in chronic fibrosis development following IR, we performed Masson's trichrome staining to measure the content of collagen fibres in lung tissues. Compared with WT mice, DNA‐PKcs^−/−^ mice showed more collagen deposition in lung tissues after IR (Figure 2A,B). Activated fibroblasts play a key role in the pathogenesis of RIPF through the synthesis and deposition of ECM.^21^, ^22^ We found that the fibroblast activation marker alpha‐smooth muscle actin (α‐SMA) was highly expressed in the lung tissues of DNA‐PKcs^−/−^ mice; moreover, collagen I accumulation was significantly increased at 5 months after IR (Figure 2C,D). These findings suggest that DNA‐PKcs deficiency contributes to fibroblast overactivation, ultimately leading to increased collagen deposition in lung tissues at the late stage of IR. Given that AECIIs have a critical function in lung tissues and can differentiate into fibroblasts via EMT, we examined the number of cells co‐expressing pulmonary surfactant protein C (SPC) and α‐SMA in mouse lung tissues by immunofluorescence staining. After IR, more SPC^+^ α‐SMA^+^ cells were detected in the lung tissues of DNA‐PKcs^−/−^ mice than in those of WT mice; this finding indicated that the deletion of DNA‐PKcs leads to a decrease in the number of AECII epithelial cells along with the accumulation of interstitial cells in lung tissue (Figure 2E). These results show that DNA‐PKcs plays a critical role in RIPF development.

### 3.3 DNA‐PKcs deficiency promoted IR‐induced EMT in vitro and in vivo

**FIGURE 3:**
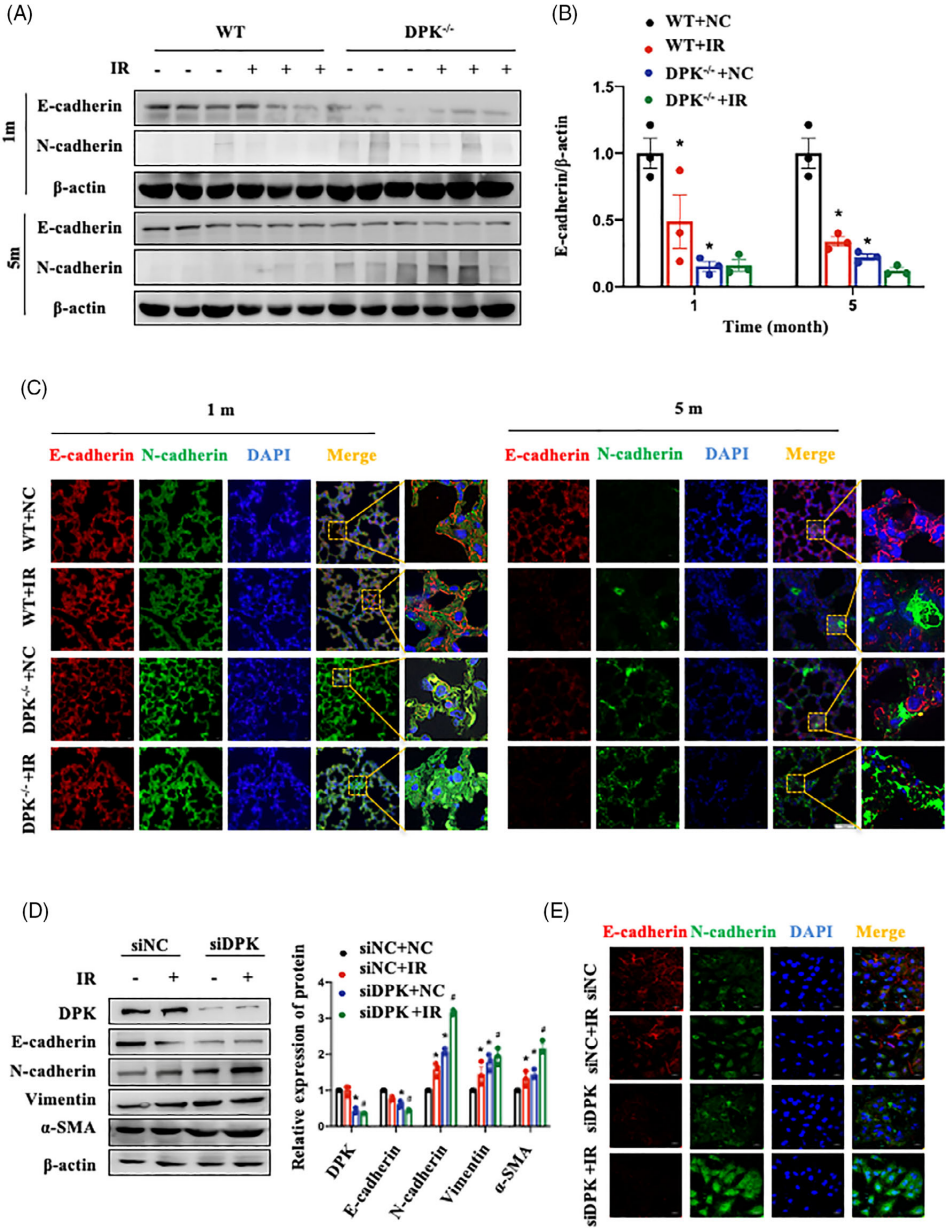
DNA‐dependent protein kinase catalytic subunit (DNA‐PKcs) deficiency promoted irradiation (IR)‐induced epithelial–mesenchymal transition (EMT) in vitro and in vivo. (A) Relative protein levels of E‐cadherin and N‐cadherin in lung tissues at 1 and 5 months after IR. Three mice per group were used for Western blotting analysis. (B) E‐cadherin expression in (A) was quantified. Data are expressed as the means ± standard error of the mean (SEM); ^*^ *p* < .05, compared with the WT + NC group; ^#^ *p *< .05, compared with the WT + IR group. (C) The localisation of E‐cadherin (shown in red) and N‐cadherin (shown in green) in lung tissues at 1 and 5 months after IR was determined by confocal microscopy. Cell nuclei were visualised by DAPI (shown in blue). Scale bar: 50 μm. (D) Relative protein levels of E‐cadherin, N‐cadherin, vimentin and α‐SMA in A549 cells with DNA‐PKcs knockdown at 48 h after IR. The experiment was repeated three times and the data are expressed as the means ± SEM; ^*^ *p* < .05, compared with the siNC + NC group; ^#^ *p *< .05, compared with the siNC + IR group. (E) Subcellular localisation of E‐cadherin (shown in red) and N‐cadherin (shown in green) in shNC and shDPK cells after IR was determined by confocal microscopy. Cell nuclei were visualised by DAPI (shown in blue). Scale bar: 20 μm. WT, wild type.

Next, we investigated whether DNA‐PKcs is involved in regulating the EMT process, a key event in PF. The results showed that DNA‐PKcs deficiency inhibited E‐cadherin expression and promoted N‐cadherin expression in the lung tissues of mice; this effect was synergistic with that of IR (Figure 3A,B). Compared to those of WT mice, the lung tissues of DNA‐PKcs^−/−^ mice exhibited a decreased number of E‐cadherin‐positive cells and an increased number of N‐cadherin‐positive cells at 1 and 5 months after IR (Figure 3C); these findings suggest that DNA‐PKcs deletion promoted the EMT phenotype. Similar results were noted in A549 human AECIIs and MLE‐12 mouse AECIIs; DNA‐PKcs inhibition increased N‐cadherin expression and decreased E‐cadherin expression (Figures 3D,E and S3A,SB). These results suggest that DNA‐PKcs deficiency promoted IR‐induced EMT in the lung tissues of mice and in AECIIs.

### 3.4 DNA‐PKcs deficiency stabilises Twist1 expression in vitro and in vivo

**FIGURE 4:**
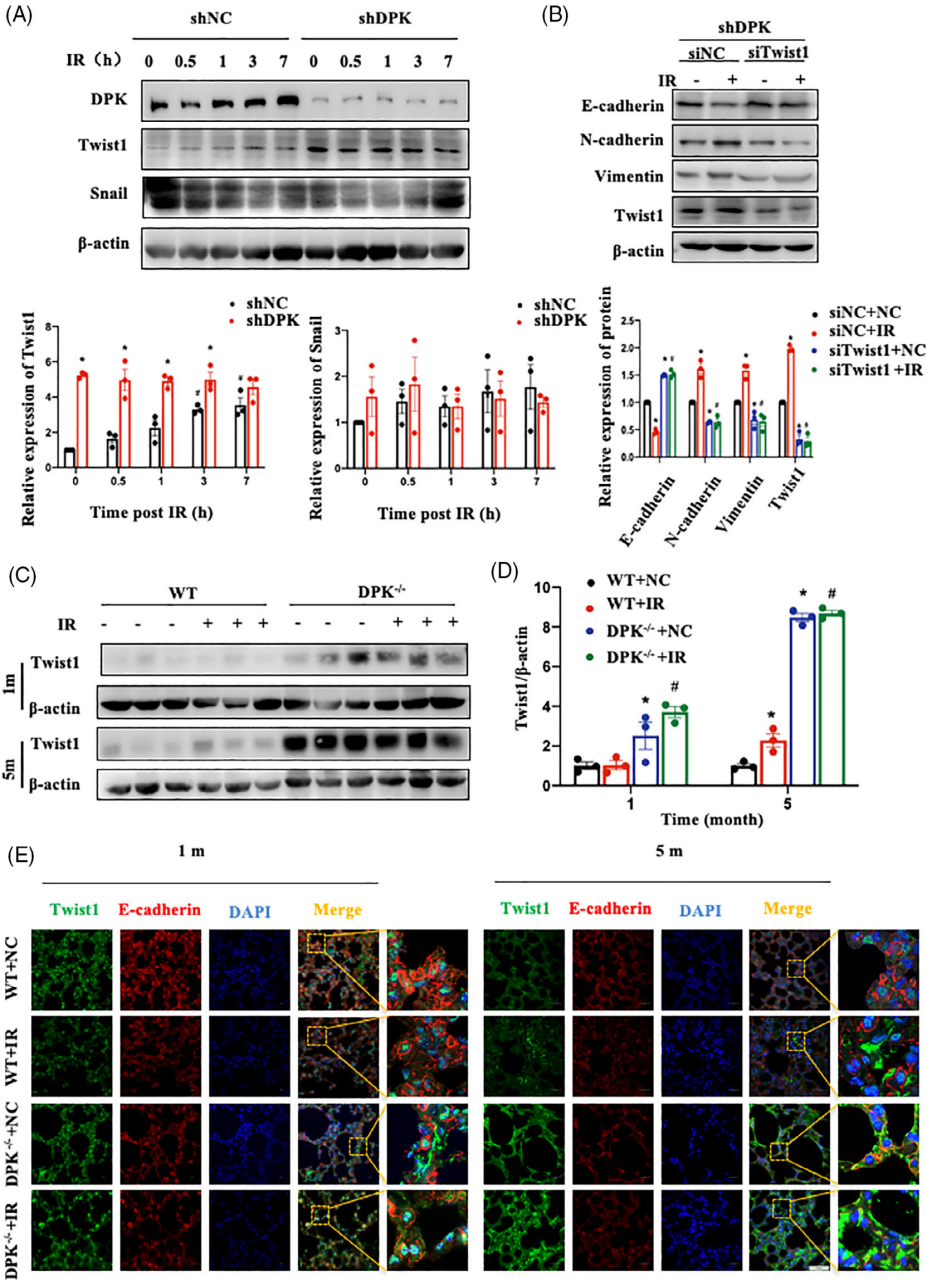
DNA‐dependent protein kinase catalytic subunit (DNA‐PKcs) deficiency increased Twist1 expression in vitro and in vivo. (A) Relative protein levels of Twist1 and Snail in DNA‐PKcs knockout cells or normal cells after irradiation (IR). The experiment was repeated three times and the data are expressed as the means ± standard error of the mean (SEM); ^*^ *p* < .05, compared with the shNC group. (B) Epithelial–mesenchymal transition (EMT)‐related protein expression in shDPK cells with Twist1 knockdown at 48 h after IR. The experiment was repeated three times and the data are expressed as the means ± SEM; ^*^ *p* < .05, compared with the siNC + NC group; ^#^ *p *< .05, compared with the siNC + IR group. (C) Relative protein levels of Twist1 in lung tissues at 1 and 5 months after IR. Three mice per group were used for Western blotting analysis. (D) Twist1 expression in (A) was quantified. Data are expressed as the means ± SEM; ^*^ *p* < .05, compared with the WT + NC group; ^#^ *p *< .05, compared with the WT + IR group. (E) The localisation of E‐cadherin (shown in red) and Twist1 (shown in green) in lung tissues at 1 and 5 months after IR was determined by confocal microscopy. Cell nuclei were visualised by DAPI (shown in blue). Scale bar: 50 μm. WT, wild type.

To clarify the mechanism by which DNA‐PKcs is involved in EMT, we examined the protein expression of key regulators of EMT, including Twist1 and Snail. DNA‐PKcs deletion specifically increased the protein content of Twist1, but expression of Snail was not regulated by changes in DNA‐PKcs expression in A549 cells after IR (Figure 4A). Moreover, Twist1 responded to IR. To determine whether DNA‐PKcs can regulate EMT in AECIIs through Twist1, we inhibited Twist1 in DNA‐PKcs‐deficient A549 cells. N‐cadherin and vimentin expression increased, and E‐cadherin expression decreased; these findings suggest that Twist1 inhibition can suppress the EMT process induced by IR in DNA‐PKcs‐deficient cells (Figure 4B). Similarly, Twist1 expression in DNA‐PKcs^−/−^ mice was considerably greater than that in WT mice, and the effect of IR on Twist1 was even masked (Figure 4C,D). Immunofluorescence assays of lung tissues revealed that an increase in Twist1 expression combined with a decrease in E‐cadherin expression after IR and DNA‐PKcs deficiency promoted this phenotype.

**FIGURE 5:**
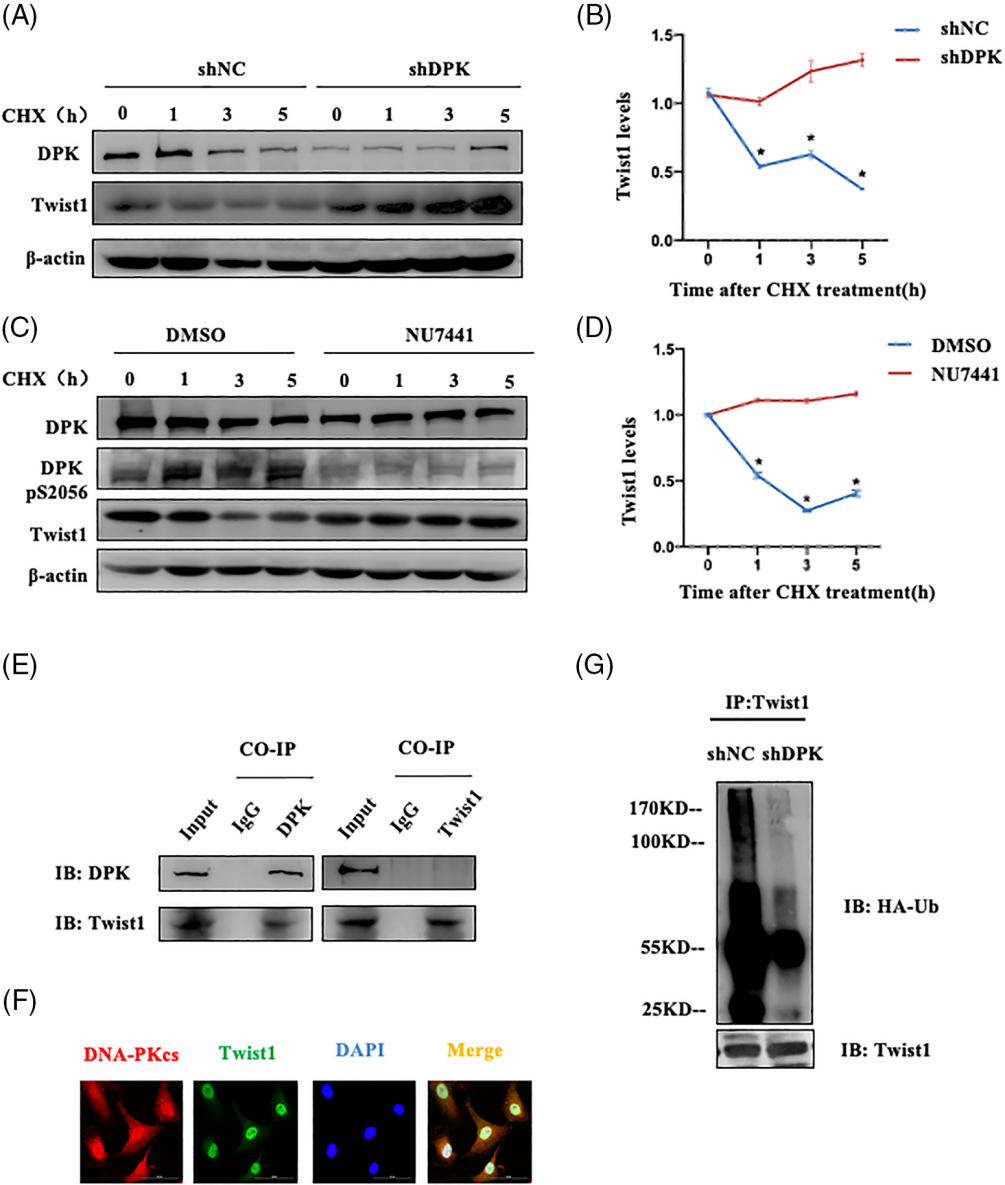
DNA‐dependent protein kinase catalytic subunit (DNA‐PKcs) mediated the ubiquitination and degradation of Twist1. (A) The half‐life of Twist1 after DNA‐PKcs knockdown was detected after cycloheximide (CHX) treatment. (B) Twist1 expression in (A) was quantified, and the value at 0 h was considered 1. The experiment was repeated three times and the data are expressed as the means ± standard error of the mean (SEM); ^*^ *p* < .05, compared with the shNC group. (C) Cells were treated with the DNA‐PKcs activity inhibitor NU7441 for 6 h. The half‐life of Twist1 was determined after CHX treatment. (D) Twist1 expression in (C) was quantified, and the value at 0 h was considered 1. The experiment was repeated three times and the data are expressed as the means ± SEM; ^*^ *p* < .05, compared with the DMSO group. (E) Co‐immunoprecipitation of DNA‐PKcs and Twist1 was performed. F. Immunofluorescence co‐localisation of DNA‐PKcs (shown in red) and Twist1 (shown in green) was performed. Cell nuclei were visualised by DAPI (shown in blue). Scale bar: 50 μm. (G) Ubiquitination of the Twist1 protein was detected in shNC and shDPK cells.

Previous studies have demonstrated that the main mechanism for protein degradation in eukaryotic cells is the ubiquitin‐mediated proteolysis pathway. Therefore, we used CHX to evaluate the degradation rate of Twist1 under conditions of DNA‐PKcs deficiency or inhibition of its kinase activity. In shDPK cells, the Twist1 degradation rate was significantly reduced, resulting in Twist1 accumulation (Figure 5A,B). Following the administration of the DNA‐PKcs kinase inhibitor NU7441, the degradation rate was also significantly inhibited (Figure 5C,D). Moreover, co‐immunoprecipitation and immunofluorescence co‐localisation assays consistently revealed an interaction between DNA‐PKcs and Twist1 in A549 cells and MLE‐12 cells (Figures 5E,F and S3C). Ubiquitination experiments revealed that DNA‐PKcs knockdown reduced Twist1 ubiquitination in A549 cells (Figure 5G). These findings suggested that DNA‐PKcs inhibition can stabilise and promote Twist1 expression, which is required for IR‐induced EMT progression.

### 3.5 DNA‐PKcs inhibition upregulated Twist1 expression by decreasing AKT1 phosphorylation in vitro and in vivo

**FIGURE 6:**
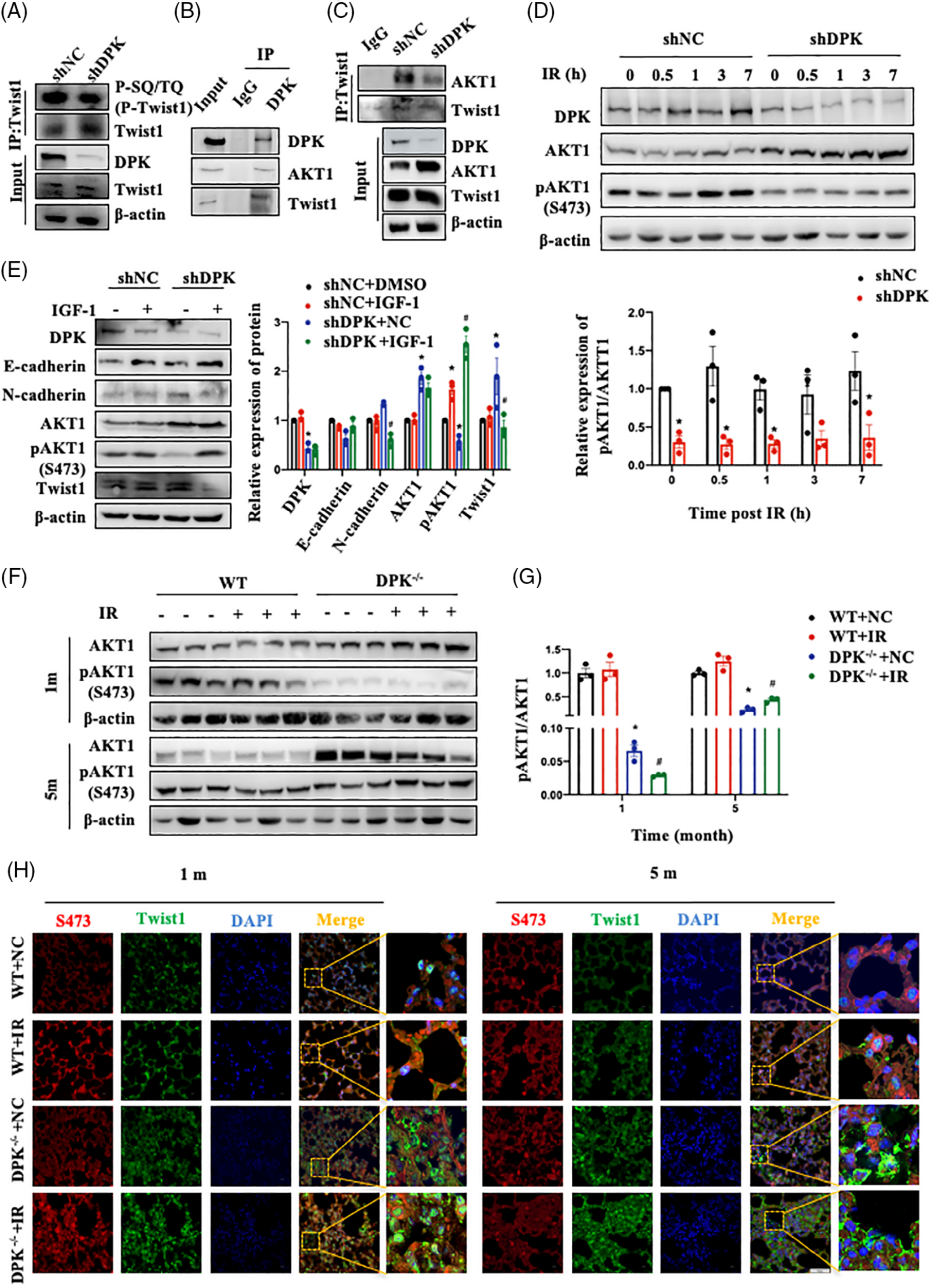
DNA‐dependent protein kinase catalytic subunit (DNA‐PKcs) inhibition upregulates Twist1 expression by decreasing AKT1 phosphorylation in vitro and in vivo. (A) Phosphorylation levels of the SQ/TQ motif on Twist1 were estimated in shNC and shDPK cells. (B) Co‐immunoprecipitation of DNA‐PKcs, AKT1 and Twist1 was performed. (C) The interaction between AKT1 and Twist1 was determined by a co‐immunoprecipitation assay in shNC and shDPK cells. (D) The phosphorylation levels of AKT1 were determined in shNC and shDPK cells at the indicated time points after irradiation (IR). The experiment was repeated three times and the data are expressed as the means ± standard error of the mean (SEM); ^*^ *p* < .05, compared with the shNC group. (E) The expression of epithelial–mesenchymal transition (EMT)‐related proteins was detected in shNC and shDPK cells after treatment with the AKT1 agonist IGF‐1 for 12 h. The experiment was repeated three times and the data are expressed as the means ± SEM; ^*^ *p* < .05, compared with the shNC + DMSO group; ^#^ *p *< .05, compared with the shNC + IGF‐1 group. (F) Phosphorylation level of AKT1 in lung tissues at 1 and 5 months after IR. Three mice per group were used for Western blotting analysis. (G) The phosphorylation level of AKT1 in (F) was quantified and is shown as the ratio of pAKT1 to AKT1. Data are expressed as the means ± SEM; ^*^ *p* < .05, compared with the WT + NC group; ^#^ *p *< .05, compared with the WT + IR group. (H) The localisation of E‐cadherin (shown in red) and Twist1 (shown in green) in lung tissues at 1 and 5 months after IR was determined by confocal microscopy. Cell nuclei were visualised by DAPI (shown in blue). Scale bar: 50 μm. WT, wild type.

A previous study showed that Twist1 phosphorylation could promote its degradation.^23^ DNA‐PKcs can phosphorylate a variety of important functional protein substrates through its serine‒threonine kinase activity, the SQ/TQ motif, and its specific recognition sequence. To confirm whether Twist1 is a direct substrate of DNA‐PKcs, we determined the level of phosphorylation of the SQ/TQ motif on Twist1. DNA‐PKcs deletion reduced the phosphorylation of the SQ/TQ motif on Twist1 (Figure 6A). We further mutated two SQ/TQ sites on Twist1 (T108A and T121A); however, these mutations did not affect their interaction (data not shown). These results suggest that DNA‐PKcs cannot directly phosphorylate Twist1. AKT1 can inhibit EMT in breast cancer cells through phosphorylation‐dependent Twist1 degradation.^23^ DNA‐PKcs interacted with AKT1 and Twist1, and DNA‐PKcs knockdown reduced the interaction between AKT1 and Twist1 in A549 cells and MLE‐12 cells (Figures 6B,C and S3D,E). DNA‐PKcs deficiency also inhibited the IR‐induced increase in AKT1 phosphorylation in A549 cells (Figure 6D). IGF‐1, an agonist of phosphorylated AKT1, reversed the increase in Twist1 and the EMT phenotype induced by DNA‐PKcs inhibition (Figure 6E); this result suggested that AKT1 is a bridge molecule through which DNA‐PKcs regulates Twist1 stability in A549 cells. AKT1 phosphorylation was decreased in the lung tissues of RILI and RIPF mice, particularly in those of DNA‐PKcs^−/−^ mice after IR (Figure 6F,G). Similar results were noted for tissue immunofluorescence staining (Figure 6H). Thus, these findings confirmed that DNA‐PKcs deficiency‐induced AKT1 inactivation affects Twist1 stability and promotes a profibrotic phenotype by enhancing EMT progression.

### 3.6 VND3207 reversed IR‐induced EMT and mitigated RIPF by enhancing DNA‐PKcs phosphorylation

**FIGURE 7:**
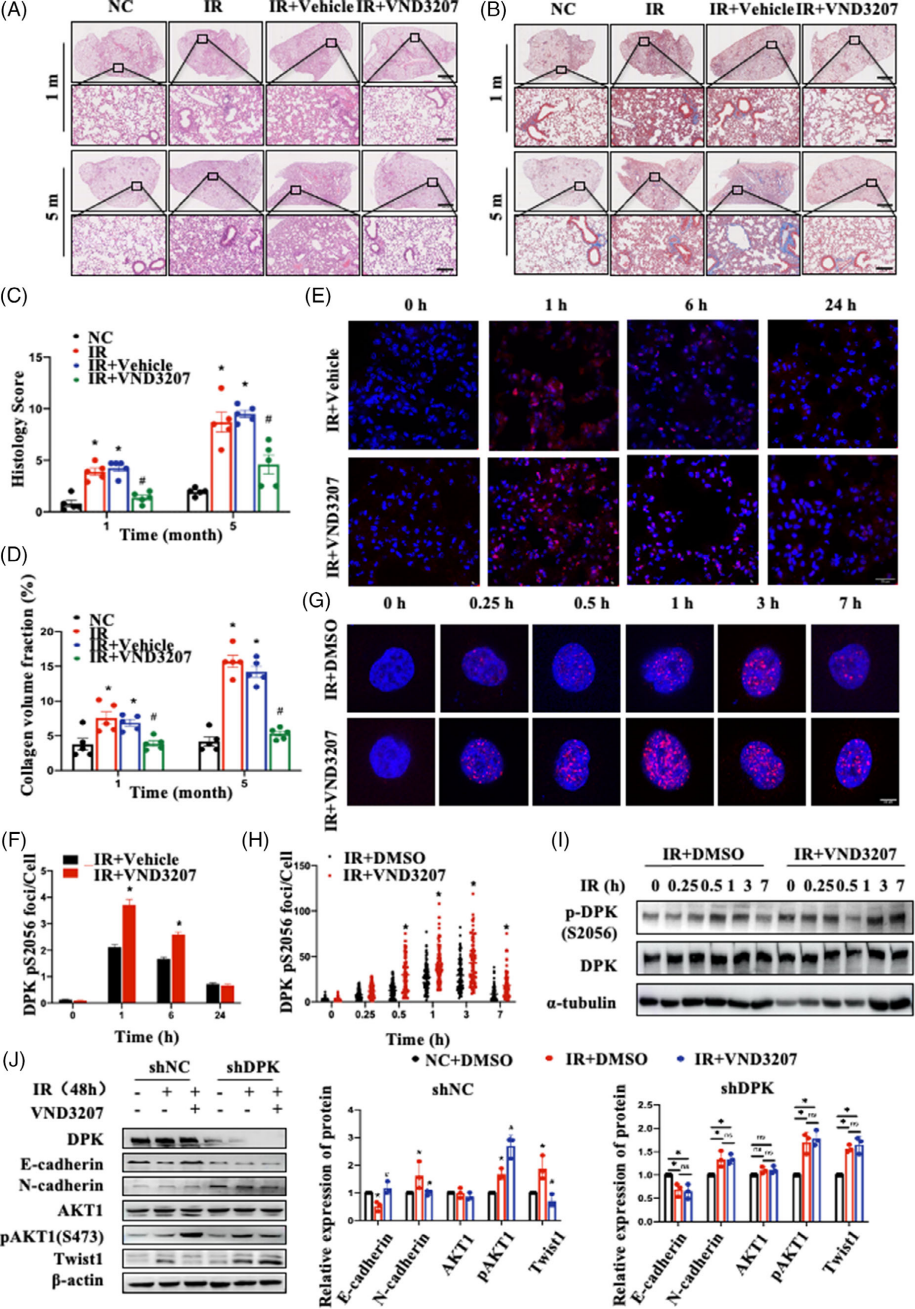
VND3207 reversed irradiation (IR)‐induced epithelial–mesenchymal transition (EMT) and mitigated radiation‐induced pulmonary fibrosis (RIPF) by enhancing DNA‐dependent protein kinase catalytic subunit (DNA‐PKcs) phosphorylation. (A) Haematoxylin‒eosin (H&E) staining of lung tissues at 1 and 5 months after IR. Scale bar: 300 or 80 μm. (B) Masson's trichrome staining of lung tissues at 1 and 5 months after IR. Scale bar: 300 or 80 μm. (C) Semi‐quantitative scoring results of lung pathology (*n* = 5 mice per group). Data are expressed as the means ± standard error of the mean (SEM); ^*^ *p* < .05, compared with the NC group; ^#^ *p* < .05, compared with the IR + vehicle group. (D) Collagen quantification results of lung tissues (*n* = 5 mice per group). Data are expressed as the mean ± SEM; ^*^ *p* < .05, compared with the NC group; ^#^ *p *< .05, compared with the IR + vehicle group. (E) Representative images of DNA‐PKcs pS2056 foci (shown in red) in lung tissues at the indicated time points after IR were analysed by confocal microscopy. Cell nuclei were visualised by DAPI (shown in blue). Scale bar: 20 μm. (F) The number of DNA‐PKcs pS2056 foci per cell at the indicated time points after IR. The foci were counted in 200 alveolar cells. Data are expressed as the mean ± SEM; ^*^ *p* < .05, compared with the IR + vehicle group. (G) Representative images of DNA‐PKcs pS2056 foci (shown in red) in A549 cells were analysed by confocal microscopy. Cell nuclei were visualised by DAPI (shown in blue). Scale bar: 10 μm. (H) The number of DNA‐PKcs pS2056 foci per cell at the indicated time points after IR. The foci of 80‒100 cells were counted. Data are expressed as the mean ± SEM; ^*^ *p* < .05, compared with the IR + vehicle group. (I) Immunoblot showing DNA‐PKcs pS2056 levels in A549 cells treated with DMSO or VND3207 at the indicated time points after IR. (J) EMT‐related protein expression in shNC and shDPK cells treated with DMSO or VND3207 at 48 h after IR. The experiment was repeated three times and the data are expressed as the mean ± SEM; ^*^ *p* < .05, compared with the NC + DMSO group; ^#^ *p *< .05, compared with the IR + DMSO group.

Our previous study reported that vanillin VND3207 is a potential radioprotective drug. This drug can protect cells against IR‐induced DNA damage. To further evaluate the therapeutic potential of VND3207 in RIPF, we administered VND3207 30 min before IR and collected lung tissues from mice for pathological observation at 1 and 5 months after IR. H&E staining revealed that VND3207 could effectively prevent the widening and rupture of the pulmonary septum caused by IR and reduce the amount of exudate in the alveolar cavity (Figure 7A,C). The results of Masson's trichrome staining showed that VND3207 could effectively reduce collagen deposition in lung tissues after IR (Figure 7B,D). These findings indicated that VND3207 could function as a preventive drug for RILI. Subsequently, we detected the phosphorylation of DNA‐PKcs at Ser2056 in lung tissues (Figure 7E,F) and A549 cells (Figure 7G,H) by an immunofluorescence assay after IR. The results revealed that the number of phosphorylated DNA‐PKcs Ser 2056 foci following VND3207 treatment was significantly greater than that in the control group. Moreover, following IR, VND3207 treatment increased and maintained the autophosphorylation of DNA‐PKcs in A549 cells (Figure 7I). We further investigated the role of VND3207 in IR‐induced EMT. The results showed that VND3207 promoted AKT1 phosphorylation and decreased Twist1 expression in shNC cells after IR but not in shDPK cells, thereby inhibiting the IR‐induced EMT process (Figure 7J). Collectively, these results indicate that VND3207 targets the DNA‐PKcs/AKT1/Twist1 axis to inhibit IR‐induced EMT and alleviate RIPF.

## 4 DISCUSSION

Radiotherapy is an important treatment approach for thoracic tumours. However, the toxicity of radiation to normal tissues is the most critical limiting factor in radiotherapy. Lung tissue is sensitive to toxicity induced by radiotherapy.^24^, ^25^ Clinical data show that approximately 16%−28% of cancer patients receiving thoracic tumour radiotherapy will develop RIPF, which is characterised by gas exchange dysfunction and gradual and irreversible destruction of lung tissue; thus, RIPF is the main dose‐limiting factor in radiotherapy.^3^ Therefore, understanding the pathogenetic mechanism of RIPF and inhibiting its progression are key approaches for improving the quality of life of patients undergoing chest radiotherapy. In the present study, we provided novel insight into the role of DNA‐PKcs in RIPF, which could be used as a potential target to prevent RIPF.

AECIIs can undergo mesenchymal cell phenotype through EMT, thus differentiate into fibroblasts to generate ECM, resulting in PF.^26^, ^27^ In the present study, we found that DNA‐PKcs decreased in human PF tissues and RIPF mice lung tissues, suggesting that DNA‐PKcs mediates PF. DNA‐PKcs deletion promoted EMT via AKT1/Twist1 regulatory in vivo and in vitro. Mechanically, DNA‐PKcs deletion inhibited the ubiquitination and degradation of Twist1. Compared to WT mice, DNA‐PKcs knockout mice showed more severe inflammatory infiltration, thickening and rupture of the pulmonary septum, and more collagen exudation following IR. Similarly, severe combined immunodeficiencies (SCID) mice lacking DNA‐PKcs activity were documented to be prone to bleomycin (BLM)‐induced PF.^28^ The result showed that BLM could induce pronounced lung eosinophilia, which reached maximal at 7 days after BLM treatment and remained elevated till 14 days in SCID mice; however, increased eosinophilia is not the absolute requirement for BLM‐induced PF in the mouse though. Moreover, with the SCID mouse model, DNA‐PKcs activity inhibition was revealed to modulate progenitor cell proliferation and fibroblast senescence to contribute IPF.^20^ Although the application of target DNA‐PKcs to prevent and therapy RIPF need further research, our results indicate that DNA‐PKcs mediates the ubiquitination and degradation of Twist1 could be a potential strategy to prevent RIPF.

In inflammatory and injured environments, EMT activates fibroblasts or other related cells for tissue reconstruction. Following the disappearance or decrease in inflammation, the EMT ceases, contributing to wound healing and tissue regeneration. EMT also persists and promotes organ fibrosis under persistent inflammation.^29^, ^30^ According to previous studies, IR can induce EMT by activating the TGF‐β, extracellular signal‐regulated kinase (ERK) and nuclear factor‐kappa B (NF‐κB) signalling pathways or by directly promoting the expression of profibrotic transcription factors such as Snail, Twist and ZEB1.^31^, ^32^ Additionally, epigenetic processes such as noncoding RNA and DNA methylation also play a critical role in IR‐induced EMT.^33^, ^34^, ^35^, ^36^, ^37^, ^38^ Although the mechanism through which radiation induces EMT has been widely discussed, the role of DNA‐PKcs in EMT remains controversial.^39^, ^40^ Our present study showed that DNA‐PKcs deletion can promote EMT in lung epithelial cells in vitro and has a synergistic effect with IR. DNA‐PKcs deletion inhibits the ubiquitination‐mediated degradation of Twist1, thereby promoting EMT. Notably, radiation can cause DNA damage, resulting in cell cycle arrest and cell senescence. Previous studies have shown that alveolar epithelial cell senescence is also a key factor in PF.^41^ Furthermore, studies have suggested that DSB repair may be involved in fibrosis.^42^, ^43^ EMT‐associated transcription factors may also regulate DSB repair,^44^, ^45^, ^46^, ^47^, ^48^ indicating that there may be crosstalk between DSB repair and EMT, and the role of DNA‐PKcs‐mediated regulation of DSB repair and senescence in alveolar epithelial cells in RIPF needs to be further elucidated in the future.

The PI3K/AKT signalling pathway is involved in regulating the EMT process in tumour cells, such as nasopharyngeal carcinoma cells, and in promoting their invasion and metastasis.^49^ As a member of the PI3K family, DNA‐PKcs can directly phosphorylate the Ser473 site in AKT1, as observed in in vitro phosphorylation experiments.^50^ The results of our in vitro and in vivo studies showed that DNA‐PKcs deletion inhibited IR‐induced AKT1 Ser473 site activation and reduced the interaction between AKT1 and Twist1. AKT1 phosphorylates Twist1 and is recognised by Trcp‐1 for ubiquitination‐based degradation.^23^ Therefore, we believe that DNA‐PKcs deletion may upregulate Twist1 expression by inhibiting AKT1 activity, thereby promoting IR‐induced EMT.

Presently, because of the lack of effective targets, there are no approved drugs for the clinical treatment of RIPF. Hence, it is important to investigate whether EMT‐targeted drugs could be used to treat RIPF. VND3207 is a vanillin derivative. Vanillin has a special aroma and is widely used as a food additive.^51^ Over the past several years, vanillin has been increasingly used in the field of medicine. Vanillin is less toxic to the body and cultured cells and has almost no genotoxicity.^52^ Previous studies have shown that vanillin can inhibit X‐ray‐ and ultraviolet‐induced chromosomal aberrations, suppress gene mutations, and reduce DNA damage.^53^, ^54^ In previous studies, we screened the vanillin derivative VND3207 and found that it has a better radioprotective effect. VND3207 was confirmed to exert no apparent toxicity to cells at a 500 μM concentration and has a wide range of radioprotective effects. VND3207 also exerts a protective effect on γ‐ray‐induced human normal lymphoblast AHH‐1 genome damage and apoptosis and can reduce DNA damage by increasing DNA‐PKcs activity.^55^ Our study revealed that VND3207 pretreatment can effectively reduce inflammatory cell infiltration, lung septal rupture and thickening and alveolar fibrin exudation in the lung tissues of mice after IR, thereby preventing RIPF progression. Mechanistically, VND3207 enhanced DNA‐PKcs activity in lung epithelial cells both in vitro and in vivo. VND3207 also reversed IR‐induced EMT by activating AKT phosphorylation in cells with normal DNA‐PKcs expression. Notably, in the DNA‐PKcs deficiency mice lung tissue there is an increase of proinflammatory cytokines (TNF‐α, IL‐6), which are also recognised as markers of the senescence‐associated secretory phenotype.^56^, ^57^ IR contributes to production of cytoplasmic free DNA fragments that can activate the cGAS/STING pathway, a key pathway involved in cellular senescence and subsequent pro‐fibrotic changes.^58^, ^59^, ^60^ Recent researches have indicated that DNA‐PKcs deletion can activate the cGAS/STING pathway.^61^, ^62^ Thus, further investigation is required to determine if VND3207 has the potential ability to modulate the DNA‐PKcs/cGAS/STING pathway to prevent RIPF. Anyway, VND3207 could be a promising drug for preventing RIPF by targeting DNA‐PKcs.

**FIGURE 8:**
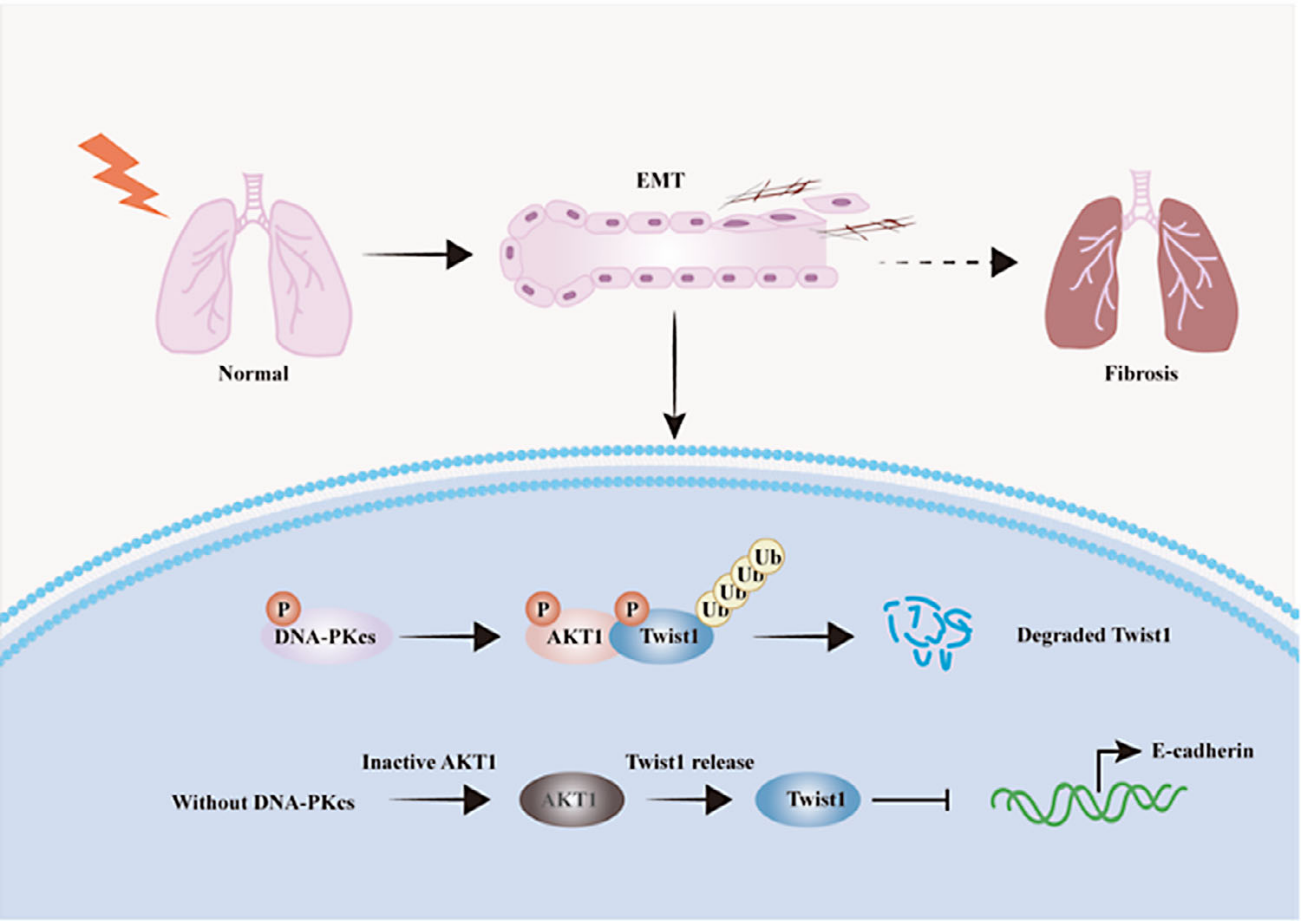
VND3207 inhibits the epithelial–mesenchymal transition (EMT) process in radiation‐induced pulmonary fibrosis (RIPF) through DNA‐PKcs‒AKT1‒Twist1 axis. DNA‐dependent protein kinase catalytic subunit (DNA‐PKcs) deficiency accelerates RIPF by inhibiting AKT1 activity and promoting Twist1 expression to enhance EMT process. The radioprotective drug VND3207 can effectively alleviate RIPF by targeting DNA‐PKcs to inhibit radiation‐induced EMT.

In conclusion (Figure 8), DNA‐PKcs deficiency accelerates EMT by inhibiting AKT1 phosphorylation and promoting Twist1 expression, leading to RIPF development. The radioprotective drug VND3207 can inhibit IR‐induced EMT by targeting DNA‐PKcs, thus effectively alleviating RIPF. The DNA‐PKcs/AKT1/Twist1/EMT in AEC II cells represents a previously unrecognised mechanism of RIPF. On the basis of these findings, we propose that DNA‐PKcs functions as a novel target for preventing RIPF by inhibiting EMT.

## AUTHOR CONTRIBUTIONS

Ziyan Yan, Jiaojiao Zhu, Yuhao Liu, Zhongqiu Li, Xinxin Liang, Shenghui Zhou, Yifan Hou, Huixi Chen, Lin Zhou, Ping Wang and Xingkun Ao performed the experiments. Ziyan Yan and Jiaojiao Zhu analysed data and wrote the manuscript. Shanshan Gao, Xin Huang, Ping‐Kun Zhou and Yongqing Gu designed the project and reviewed and edited manuscript. All the authors contributed to the interpretation of the results and the proof reading of the manuscript.

## CONFLICT OF INTEREST STATEMENT

The authors declare they have no conflicts of interest.

## ETHICS STATEMENT

Animal experiments were conducted in accordance with the Laboratory Animal Guideline of Welfare and Ethics of China and All experimental procedures were approved by the Animal Care and Use Committee at the Military Academy of Medical Sciences (IACUC‐DWZX‐2020‐792).

## CONSENT FOR PUBLICATION

All authors have read and approved the final version of the manuscript.

## Supporting information

Supporting Information

Supporting Information

Supporting Information

Supporting Information

## Data Availability

The data are available on the request.
