# Supplementary material for: DNA‐PKcs/AKT1 inhibits epithelial–mesenchymal transition during radiation‐induced pulmonary fibrosis by inducing ubiquitination and degradation of Twist1
Source: Clin Transl Med. 2024 May 17;14(5):e1690. doi: 10.1002/ctm2.1690 (PMC11101672; doi:10.1002/ctm2.1690)
Supplement: Supplementary file 1 — Supporting Information [file CTM2-14-e1690-s003.docx]

**Supplementary Table 1. The primer sequences for RT-PCR**

| m-TNF-α-F | 5’- CGAGTGACAAGCCTGTAGCCC -3’ |
| --- | --- |
| m-TNF-α-R | 5’- GTCTTTGAGATCCATGCCGTTG -3’ |
| m-IL-1β-F | 5’- CTTCAGGCAGGCAGTATCACTC -3’ |
| m-IL-1β-R | 5’- TGCAGTTGTCTAATGGGAACGT -3’ |
| m-IL-6-F | 5’- ACAACCACGGCCTTCCCTAC -3’ |
| m-IL-6-R | 5’- TCTCATTTCCACGATTTCCCAG -3’ |
| m-TGF-β-F | 5’- AGACCACATCAGCATTGAGTG -3’ |
| m-TGF-β-R | 5’- GGTGGCAACGAATGTAGCTGT -3’ |
| m-β-actin-F | 5’- TGCTGTCCCTGTATGCCTCT -3’ |
| m-β-actin-R | 5’- TTTGATGTCACGCACGATTT -3’ |
| m, mouse；F, forward；R, reverse | |

**Supplementary Table 2. The primary antibodies used in the experiment**

| Antibodies | Source | Identifier |
| --- | --- | --- |
| anti-E-cadherin | Cell Signaling Technology, MA, USA | Cat#3195S |
| anti-N-cadherin | Cell Signaling Technology, MA, USA | Cat#14215S and 13116S |
| anti-Collagen I | Proteintech, Wuhan, China | Cat#14695-1-AP |
| anti-α-SMA | Abcam, MA, USA | Cat#ab124964 |
| anti-Vimentin | Abcam, MA, USA | Cat#ab8978 |
| anti-Twist1 | Abcam, MA, USA  Proteintech, Wuhan, China | Cat#ab175430  Cat#25465-1-AP |
| anti-DNA-PKcs | Invitrogen, Shanghai, China  Santa Cruz, CA, USA | Cat#MA5-13238  Cat#sc-390849 |
| anti-phospho-DNA-PKcs（Ser2056） | Abcam, MA, USA  Signalway antibody, MD, USA | Cat#Ab174576  Cat#13918 |
| anti-AKT1 | Proteintech, Wuhan, China | Cat#10176-2-AP |
| anti-phospho-AKT1(Ser473) | Abcam, MA, USA | Cat#81283 |
| anti-Myc-tag (HRP) | Abcam, MA, USA | Cat#ab62928 |
| anti-SPC | Abcam, MA, USA | Cat#ab124964 |
| anti-β-actin | ZSGB-BIO, Beijing, China | Cat#TA-09 |
| anti-GAPDH | ZSGB-BIO, Beijing, China, | Cat#TA-08 |

**Supplementary Figure 1. The activity of DNA-PKcs in RIPF mice lung tissues.** A. The activity of DNA-PKcs in lung tissues by immunohistochemical staining. B. The expression and activity (ser2056) of DNA-PKcs in lung tissues by western blotting. Three mice per group were used for western blot analysis. C. The localization of γ-H2AX (shown in green) and DNA-PKcs ser2056 (shown in red) in lung tissues at 5 months after IR was determined by confocal microscopy. Cell nuclei were visualized by DAPI (shown in blue). White arrows indicate nuclear γ-H2AX foci. Scale bar, 50 μm. D. The number of γ-H2AX foci per cell in lung tissues at 5 months after IR. The foci were counted in more than 100 cells. Data are expressed as the means ± SEMs; ^*^*P* < 0.05, compared with the NC group.

**Supplementary Figure 2. Construction and identification of DNA-PKcs knockout mice.** A. DNA sequence alignment of exon 1 in wild-type mice (top) and DNA-PKcs-knockout mice (bottom). B. DNA sequence alignment of exon 12 in wild-type mice (top) and DNA-PKcs-knockout mice (bottom). DNA-PKcs is encoded by the PRKDC gene. C. DNA-PKcs expression in lung tissues of wild-type mice (WT) and DNA-PKcs-knockout mice (DPK^-/-^). Three mice per group were used for western blot analysis.

**Supplementary Figure 3. DNA-PKcs inhibition upregulates Twist1 expression by decreasing AKT1 phosphorylation in MLE-12 cells.** A. EMT-related protein expression in MLE-12 cells after DNA-PKcs knockdown. The experiment was repeated three times and the data are expressed as the means ± SEMs; ^*^ *P* < 0.05, compared with siNC group. B. EMT-related protein expression in MLE-12 cells after DNA-PKcs knockdown and IR. The experiment was repeated three times and the data are expressed as the means ± SEMs; ^*^ *P* < 0.05, compared with siNC+NC group, ^#^ *P* < 0.05, compared with siNC + IR group. C. Immunofluorescence co-localization of DNA-PKcs (showed in red) and Twist1 (showed in green) was performed in MLE-12 cells, Cell nuclei were visualized by DAPI (showed in blue). Scale bar, 50 μm. D. The interaction between DNA-PKcs, AKT1 and Twist1 in MLE-12 cells. E. The interaction between AKT1 and Twist1 in MLE-12 cells after DNA-PKcs knockdown.
