# Supplementary figures and images for: DNA‐PKcs/AKT1 inhibits epithelial–mesenchymal transition during radiation‐induced pulmonary fibrosis by inducing ubiquitination and degradation of Twist1
Source: Clin Transl Med. 2024 May 17;14(5):e1690. doi: 10.1002/ctm2.1690 (PMC11101672; doi:10.1002/ctm2.1690)

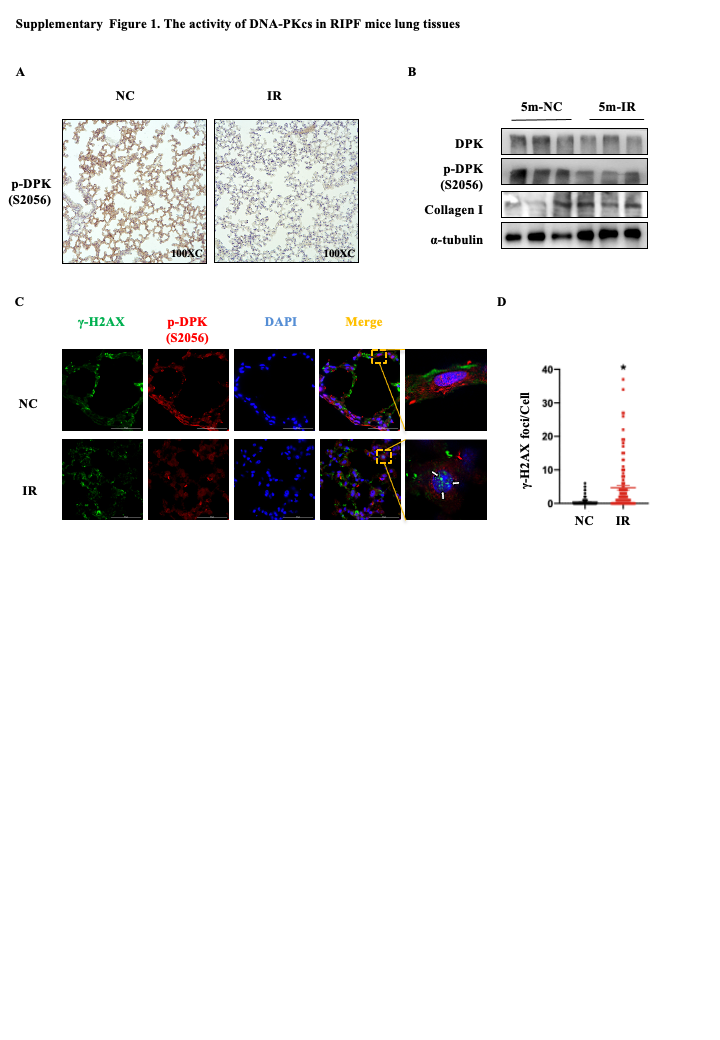

Supplement: Supplementary file 2 — Supporting Information [file CTM2-14-e1690-s001.tiff]

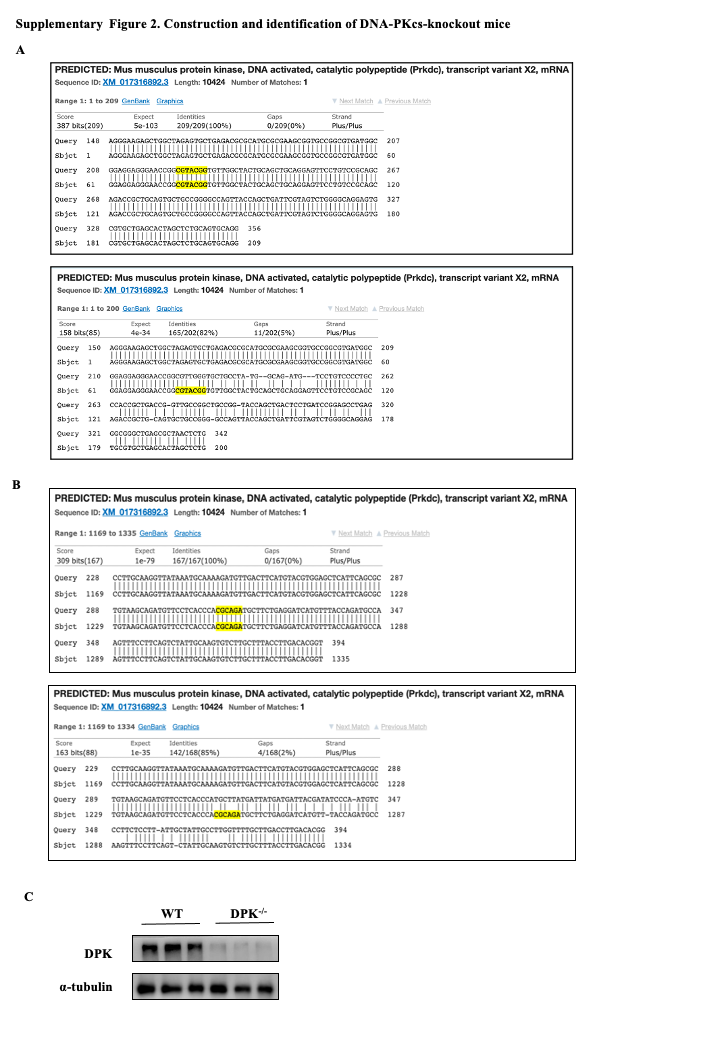

Supplement: Supplementary file 3 — Supporting Information [file CTM2-14-e1690-s004.tiff]

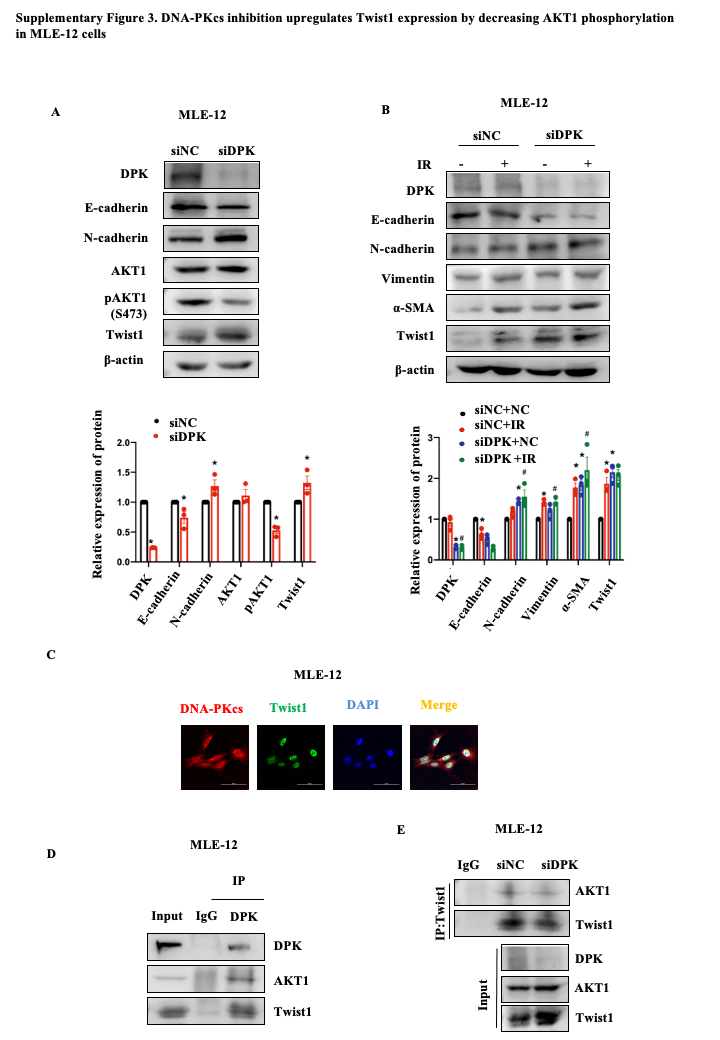

Supplement: Supplementary file 4 — Supporting Information [file CTM2-14-e1690-s002.tiff]
